# Sprint cycling rate of torque development associates with strength measurement in trained cyclists

**DOI:** 10.1007/s00421-023-05143-1

**Published:** 2023-02-10

**Authors:** Shannon Connolly, Peter Peeling, Martyn J. Binnie, Paul S. R. Goods, Christopher Latella, Janet L. Taylor, Anthony J. Blazevich, Wouter P. Timmerman, Chris R. Abbiss

**Affiliations:** 1 Centre for Human Performance, School of Medical and Health Sciences, Edith Cowan University, Perth, WA Australia; 2 Western Australian Institute of Sport, Mount Claremont, Perth, WA Australia; 3 School of Human Sciences (Exercise and Sport Science), The University of Western Australia, Crawley, Perth, WA Australia; 4 Murdoch Applied Sports Science Laboratory, Murdoch University, Perth, WA Australia; 5 Centre for Healthy Ageing, Health Futures Institute, Murdoch University, Perth, WA Australia; 6 Neurophysiology Research Laboratory, Edith Cowan University, Perth, WA Australia

**Keywords:** Cycling, Force development, Peak force, Performance testing, Strength

## Abstract

**Purpose:**

A cyclist’s rate of force/torque development (RFD/RTD) and peak force/torque can be measured during single-joint or whole-body isometric tests, or during cycling. However, there is limited understanding of the relationship between these measures, and of the mechanisms that contribute to each measure. Therefore, we examined the: (i) relationship between quadriceps central and peripheral neuromuscular function with RFD/RTD in isometric knee extension, isometric mid-thigh pull (IMTP), and sprint cycling; and (ii) relationship among RFD/RTD and peak force/torque between protocols.

**Methods:**

Eighteen trained cyclists completed two familiarisation and two experimental sessions. Each session involved an isometric knee extension, IMTP, and sprint cycling protocol, where peak force/torque, average and peak RFD/RTD, and early (0–100 ms) and late (0–200 ms) RFD/RTD were measured. Additionally, measures of quadriceps central and peripheral neuromuscular function were assessed during the knee extension.

**Results:**

Strong relationships were observed between quadriceps early EMG activity (EMG_50_/M) and knee extension RTD (*r* or *ρ* = 0.51–0.65) and IMTP late RFD (*r* = 0.51), and between cycling early or late RTD and peak twitch torque (*r* or *ρ* = 0.70–0.75). Strong-to-very strong relationships were observed between knee extension, IMTP, and sprint cycling for peak force/torque, early and late RFD/RTD, and peak RFD/RTD (*r* or *ρ* = 0.59–0.80).

**Conclusion:**

In trained cyclists, knee extension RTD or IMTP late RFD are related to measures of quadriceps central neuromuscular function, while cycling RTD is related to measures of quadriceps peripheral neuromuscular function. Further, the strong associations among force/torque measures between tasks indicate a level of transferability across tasks.

**Supplementary Information:**

The online version contains supplementary material available at 10.1007/s00421-023-05143-1.

## Introduction

The magnitude and rate at which a cyclist can apply force to the pedal to produce bicycle crank torque are important factors dictating sprint cycling performance (Gardner et al. 2007; Watsford et al. 2010), which is emphasised by the increased use of systematic resistance training by sprint cyclists (Munro and Haff 2018). Maximal torque production is important in overcoming inertia to accelerate the bicycle forwards, while the rate at which torque is applied to the crank is critical given the limited time available for torque production when cycling at moderate-to-high cadences. For instance, when pedalling at cadences of 80 revolutions per minute (rpm) or higher, the time available for muscular force production during the pedal downstroke can be less than the 300 ms needed to produce maximal torque in important lower limb muscles such as the knee extensors during an explosive maximal voluntary isometric contraction (MVIC) protocol (Aagaard et al. 2002). Consequently, a greater mechanical crank rate of torque development (RTD) early (i.e., first 100 ms) in the pedal downstroke will result in a steeper torque rise and thus greater impulse, which will thus increase mean crank power and bicycle speed.

To date, evidence for the physiological and biomechanical factors contributing to RTD is mostly limited to studies using single-joint tasks (Del Vecchio et al. 2019; Andersen and Aagaard 2006; Maffiuletti et al. 2016; Cossich and Maffiuletti 2020). While these studies have documented that early (≤ 100 ms from contraction onset) and late (≥ 200 ms from contraction onset) torque development capacities are strongly related to neural activation transmitted by the motor neurons (i.e., central neuromuscular function) and muscular factors (i.e., peripheral neuromuscular function), respectively, this relationship has yet to be investigated in single-joint and whole-body tasks in cyclists. Accordingly, further research is required to assess the relationship between measures of central and peripheral neuromuscular function in an isolated single-joint assessment and the rate of force development (RFD) and RTD in single-joint and whole-body tasks. Such an investigation is important to determine whether RFD/RTD measurement in single-joint and whole-body tasks can provide an indication of central or peripheral neuromuscular function when techniques such as electromyography (EMG) or tetanic muscle stimulation are not available.

Cyclist neuromuscular function is commonly assessed using laboratory, gym, and field-based performance tests (Stone et al. 2004; Gardner et al. 2007; Kordi et al. 2020; Wackwitz et al. 2021). Isolated single-joint tasks such as knee extension have traditionally been used within laboratory-based research (Aagaard et al. 2002). While these assessments allow a controlled and comprehensive evaluation of neuromuscular function (Maffiuletti et al. 2016), they are mechanically different to the whole-body dynamic tasks inherent to most sports (Baker et al. 1994). Neuromuscular function can also be assessed in a gym-based environment using whole-body isometric tests such as the isometric mid-thigh pull (IMTP) (Guppy et al. 2022). Although gym-based isometric tests have been proposed to be safer (from an injury perspective) and more time-efficient than other dynamic assessments such as maximal-effort (e.g., one-repetition maximum) squat lifts, jumps, or throws (Guppy et al. 2018), measurement of neuromuscular function during whole-body isometric tasks does not replicate the dynamic movement patterns and task specificity of sporting tasks. Conversely, whole-body dynamic tests can provide a sports-specific assessment of neuromuscular function. However, these assessments provide limited information regarding the central and peripheral contributions to neuromuscular function.

Of note, research investigating relationships among RFD/RTD and peak torque between single-joint knee extension, whole-body IMTP, and whole-body dynamic cycling assessments of neuromuscular function is absent in trained or well-trained cyclists. A notable biomechanical difference in the production of force/torque during these exercises is the relative contribution of the quadricep muscles. This ranges from the isolation of the knee extensors for the knee extension task (Maffiuletti et al. 2016), to the multi-joint (and thus multi-muscle) isometric nature of the IMTP, to the compound movement of sprint cycling using all the major muscle groups in the lower limbs to produce impulse at the pedal (Raasch et al. 1997; Dorel et al. 2012, McDaniel et al. 2014). While researchers have reported the relationships between knee extension and sprint cycling peak torque (Driss et al. 2002; *r* = 0.73) or quadriceps muscle activation (Dorel et al. 2012) in trained or elite cyclists, and between IMTP peak force and sprint cycling peak torque in trained cyclists (Vercoe and McGuigan 2018; *r* = 0.93), no studies have investigated the relationship between RFD/RTD measures in single-joint, and whole-body isometric tests and in sprint cycling in trained or well-trained cyclists. Knowledge in this area is important to better understand the relationships between measures obtained in common laboratory, gym, and sports-specific tests.

Therefore, the aims of the present study were to: (i) examine the relationships between measures of quadriceps central and peripheral neuromuscular function assessed in an isometric knee extension test and RFD/RTD in knee extension, IMTP, and sprint cycling; and (ii) investigate the relationships among RFD/RTD, and peak force/torque between knee extension, IMTP, and sprint cycling. Based on the findings of Driss et al. (2002) and Vercoe and McGuigan (2018), we hypothesised that there would be a strong relationship among RFD/RTD, and peak force/torque between the three protocols. In addition, given the previously documented strong relationship between cycling peak power and quadriceps muscle volume (*r* = 0.81; *p* < 0.001) or vastus lateralis pennation angle (*r* = 0.81; *p* < 0.001) (Kordi et al. 2020), we hypothesised that cycling RTD would have the strongest correlation with quadriceps peripheral neuromuscular function measures.

## Methodology

### Participants

Three trained and fifteen well-trained cyclists (McKay et al. 2022) (*n* = 13 men, 5 women; age 28 ± 9 years, height 174.4 ± 8.2 cm, body mass 76.3 ± 10.6 kg, habitual training 10.4 ± 6.0 h week^−1^) volunteered for this study. The inclusion criteria were that participants were classified as at least ‘trained’ cyclists using the participant classification framework set by McKay et al. (2022). Participants were excluded if they presented with adverse cardiovascular or musculoskeletal risk factors or had incomplete datasets. Following data collection, the IMTP datasets for two participants were incomplete, and thus, data for 16 participants are presented for this protocol (and any correlations with IMTP variables). Data for 18 participants are presented for the cycling and knee extension protocols (as above, data for 16 presented when correlated with IMTP variables). Prior to study commencement, participants provided written informed consent. Ethics approval was provided by the host institution’s Ethics Committee.

### Experimental overview

Participants attended two familiarisation sessions prior to two experimental sessions. The familiarisation sessions allowed participants to become accustomed to and complete the test protocols in full. Anthropometric data (height, body mass) were also collected, and the equipment set-up determined. Testing sessions were separated by 5 ± 3 days. Each session commenced at the same time of the day (± 1 h) to avoid diurnal fluctuations in performance (Teo et al. 2011). During all sessions, participants completed the same knee extension, IMTP, and sprint cycle protocols, which were separated by 30 min passive rest (Floyd et al. 2013). The order of the protocols and the set order (for the knee extension and IMTP) were randomised during the first familiarisation session and standardised throughout. Before all visits, participants were requested to refrain from ingesting stimulants or depressants for 12 h, strenuous exercise for 24 h, and to arrive 3 h post-prandial in a well-hydrated state.

### Experimental procedures

#### Isometric Mid-thigh Pull (IMTP)

Before the maximal IMTP testing, participants performed a warm-up consisting of dynamic movements (e.g., body weight squat, lunges), one set of three submaximal dynamic mid-thigh pulls, and one set of three submaximal IMTPs of increasing intensity (Guppy et al. 2022). Following 2 min of passive rest, participants were placed in a posture and bar position corresponding to the start of the second pull of the power clean (Haff et al. 1997) with hip and knee angles of 146.1 ± 4.4° and 141.7 ± 3.4°, respectively. Participants then performed one set of five 1-s and one set of five 5-s IMTP trials, with 1-min passive rest between trials and 10-min passive rest between sets. Participants were instructed to complete each trial ‘as fast and as hard as possible’ for the 1-s trials, and as ‘hard and as fast as possible’ for the 5-s trials (Guppy et al. 2022). All IMTP trials were performed in a custom-designed power rack (Fitness Technology, Adelaide, Australia) which allowed for the barbell to be positioned at any height above a force plate (BP12001200, AMTI, Newton, MA) through a combination of pins and hydraulic jacks. After being set to the correct height, the bar was further secured by clamps to ensure that any movement of the system upon force application was as minimal as possible (Maffiuletti et al. 2016). Vertical ground reaction forces were collected at 2000 Hz using custom LabVIEW software (version 14.0; National Instruments, TX, USA) via a BNC-2090 interface box with an analog-to-digital card (NI-6014, National Instruments, TX, USA). The standardisation of the set-up (i.e., joint angles, bar height, hand grip width, foot position), individual trial countdown, implementation of the pull, and in-session trial exclusion criteria replicated the methods described previously by Guppy et al. (2022).

**Fig. 1:**
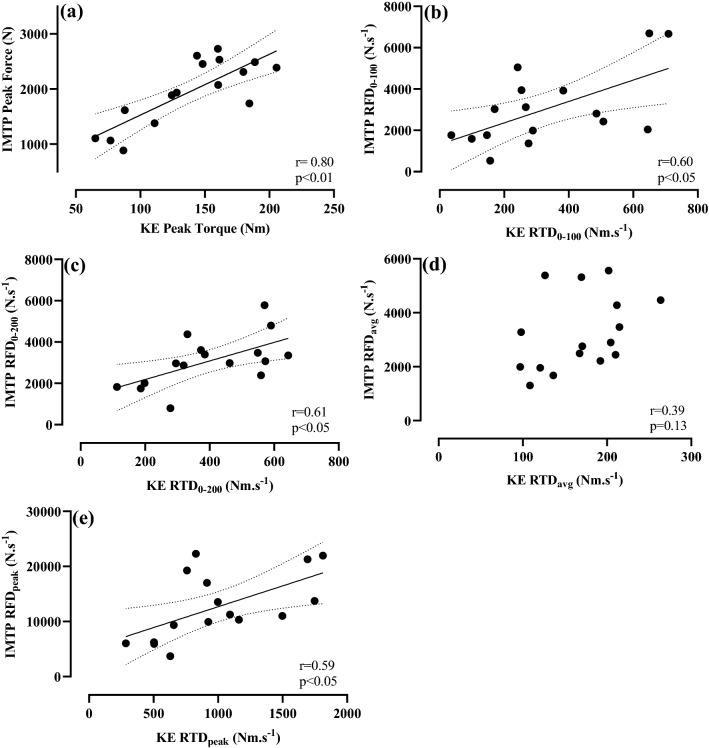
Pearson’s correlation coefficients (*r*) between isometric mid-thigh pull (IMTP) and knee extension (KE): **a** peak force/torque, **b** rate of force/torque development from 0 to 100 ms (RFD/RTD_0–100_), **c** RFD/RTD from 0 to 200 ms (RFD/RTD_0–200_), **d** average RFD/RTD (RFD/RTD_avg_), and **e** peak RFD/RTD (RFD/RTD_peak_). Dotted lines: 95% confidence intervals

No filtering was applied to the force–time data during analysis (Dos’Santos et al. 2018). All collected force–time curves were analysed using custom LabVIEW software (Version 14.0, National Instruments). Force onset was defined as ‘*the last peak/trough before the signal deflects away from baseline noise’* (Tillin et al. 2010) and identified manually using previously outlined methods (Guppy et al. 2021) (Supplementary Fig. 1). Force (or torque) onset determination was made by the lead investigator for all protocols in this study. The maximum force during each trial was reported as the peak force (PF). Peak RFD (RFD_peak_) was the fastest RFD during any 20 ms sampling window (Haff et al. 2015). Early and late RFD (or RTD for protocols below) were defined as RFD in the time bands 0–100 ms (RFD_0–100_) and 0–200 ms (RFD_0–200_), respectively, with both calculated as the quotient of the changes in force and time. Average RFD (RFD_avg_) was calculated as the change in force from force onset to PF divided by the time elapsed (Haff et al. 2015). In accordance with previous research (Guppy et al. 2022), RFD_0–100_, RFD_0–200_, RFD_avg_ and RFD_peak_ were significantly greater (p < 0.05) in the 1-s than the 5-s IMTP, and thus, the IMTP data presented for these variables (Figs. 1 and 3) were derived from the 1-s IMTP tests. PF was not statistically different in the 1-s and 5-s IMTP, and thus, IMTP PF data presented (Figs. 1 and 3) were derived from the 5-s IMTP in line with previous recommendations (Guppy et al. 2022). The PF was reported as the maximum force minus the participant’s body weight. Once processed, the means of the three ‘best trials’ within each set (i.e., 1 set of five 1-s, 1 set of five 5-s) in each testing session were used for statistical analysis. The ‘best trials’ were defined as the trials with the greatest peak force for PF processing, or the trials with the highest RFD_0–200_ for the processing of RFD variables (Guppy et al. 2021).

### Knee extension and electrical stimulation procedures

Single-joint neuromuscular function was assessed during an explosive knee extension maximal voluntary isometric contraction (MVIC) protocol. Participants were seated upright in a custom-built chair (80/20 Australia, NSW) with their preferred leg for starting a cycling sprint secured to an in-line force transducer (UU-K100 100 kg, Load cell, Australia) via a velcro™ strap 2 cm above the ankle and rope attached to an immovable bar behind the leg. Another velcro™ strap and rope attached to an anterior immovable bar was used to suspend the leg with the hip and knee flexed to 90° (0° = full extension) (Boccia et al. 2016). Hip and knee angles were both confirmed by hand-held goniometry and then maintained for each subject across all sessions. Participants were fitted with a waist strap to minimise extraneous trunk movement, and chair set-up was measured and standardised for all sessions. EMG of vastus lateralis (VL), vastus medialis (VM), and rectus femoris (RF) were recorded with surface electrodes (Ag–AgCL) in monopolar configuration, with one electrode positioned over the muscle belly and the other placed ~ 5 cm distal, and a ground placed over the tibial tuberosity. Recording electrodes for the VL, VM, and RF were placed at 66, 80, and 50% of the distance between the inguinal crease and the top of the patella, respectively. Before electrode placement, skin was cleaned with 70% isopropyl alcohol swabs. The EMG signals were amplified (1000×) and filtered (20 Hz–1 kHz; CED 1902 amplifier, Cambridge Electronic Designs) and both EMG and knee extensor force signals were digitised at 2 kHz (CED 1401, Cambridge Electronic Design, UK) (Table 1).

**Table 1:** Values are mean ± SD

Protocol	Metric	Session 1	Session 2
Cycling	*T*_peak_ (Nm)	251 ± 48	251 ± 48
	*T*_0_ (Nm)	195 ± 42	194 ± 46
	RTD_0–100_ (Nm s^−1^)	562 ± 220	603 ± 249
	RTD_0–200_ (Nm s^−1^)	858 ± 276	871 ± 288
	RTD_avg_ (Nm s^−1^)	803 ± 250	802 ± 263
	RTD_peak_ (Nm s^−1^)	1389 ± 451	1380 ± 484
Isometric mid-thigh pull	Peak force (N)	2695 ± 676	2686 ± 681
	RFD_0–100_ (N s^−1^)	3060 ± 1633	3030 ± 2017
	RFD_0–200_ (N s^−1^)	3181 ± 1159	3001 ± 1336
	RFD_avg_ (N s^−1^)	3306 ± 1342	3131 ± 1542
	RFD_peak_ (N s^−1^)	12,710 ± 5898	12,657 ± 6380
Knee extension	Peak torque (Nm)	138 ± 43	142 ± 48
	RTD_0–100_ (Nm s^−1^)	302 ± 196	344 ± 235
	RTD_0–200_ (Nm s^−1^)	395 ± 166	413 ± 170
	RTD_avg_ (Nm s^−1^)	179 ± 59	165 ± 44
	RTD_peak_ (Nm s^−1^)	963 ± 422	1037 ± 528
	Ttw,p (Nm)	77 ± 23	77 ± 24
	VA (%)	80 ± 8	80 ± 6
	20:80 (Nm)	0.68 ± 0.08	0.68 ± 0.07
	20:VFT (Nm)	0.98 ± 0.03	0.98 ± 0.03
	*t*_tw,p_ (s)	0.12 ± 0.02	0.12 ± 0.02
	*t*_1/2_ (s)	0.07 ± 0.02	0.08 ± 0.01
	VL EMG_50_/M (mV)	0.03 ± 0.03	0.03 ± 0.02
	VM EMG_50_/M (mV)	0.03 ± 0.01	0.03 ± 0.01
	RF EMG_50_/M (mV)	0.02 ± 0.01	0.03 ± 0.01
	Average EMG_50_/M (mV)	0.03 ± 0.02	0.03 ± 0.01

Cycling, isometric mid-thigh pull (IMTP) and knee extension (KE) neuromuscular function measures for experimental sessions 1 and 2. Observed peak torque (*T*_peak_); theoretical peak torque (*T*_0_); rate of force/torque development (RFD/RTD) from 0 to 100 ms (RFD/RTD_0–100_); RFD/RTD from 0 to 200 ms (RFD/RTD_0–200_); average RFD/RTD (RFD/RTD_avg_); peak RFD/RTD (RFD/RTD_peak_). Vastus lateralis, vastus medialis, and rectus femoris electromyograph amplitude to 50 ms normalised to M-wave (VL, VM, and RF EMG_50_/M); Average EMG_50_/M is VL + VM + RF/3; voluntary activation (VA%); ratio of torques evoked by 20-Hz and 80-Hz stimulations (20:80); ratio of torques evoked by 20 Hz and variable-frequency train (20:VFT) stimulations; peak twitch torque (*Τ*_tw,p_); time to peak twitch (*t*_tw,p_); and peak twitch half relaxation time (*t*_1/2_)

Before knee extension MVIC testing, the maximum muscle compound action potential (*M*_max_) was determined by manual identification and stimulation of the femoral nerve using procedures previously described by Barley et al. (2018). The stimulus intensity used to elicit the *M*_max_ was increased by 20% (Tillin et al. 2010) for subsequent testing to ensure a supramaximal stimulus intensity to account for possible depression of motor responses during the MVICs.

Before testing, participants performed a warm-up involving eight brief voluntary knee extensor contractions beginning at 30% of perceived MVIC and progressively increasing until reaching 100% of perceived MVIC for the final contraction (Barley et al. 2018). A 2-min rest was given before the testing commenced. Participants completed the same set and effort durations (i.e., 1 set of five 1-s, 1 set of five 5-s), with the same rest time, and followed the same instructions for 1-s and 5-s efforts as described for the IMTP protocol above. Two electrical stimuli (100 Hz doublet, 0.2-ms duration) were delivered when peak force was visually identified during the 5-s efforts and another stimulus was delivered 2–3 s later during rest using a constant-current stimulator (Digitimer DS7AH, UK). The twitch responses from both sets of stimuli were used to calculate voluntary activation (VA%) for each contraction using a correction equation (Strojnik and Komi 1998). Peak twitch torques (*Τ*_tw,p_), times to peak twitch torque (*t*_tw,p_), and peak twitch half relaxation times (*t*_1/2_) were measured from the resting twitch 2–3 s after the MVIC.

Tetanic muscle stimulations were completed 10 min after the MVIC protocol to assess excitation–contraction (E–C) coupling efficiency of the knee extensor muscles. Electrical square-wave stimuli (0.5-ms pulse width) were delivered to the knee extensor muscle belly through four self-adhesive electrodes (5 × 9 cm, Dura-stick II, Chattanooga group, Hixson, TN, United States) using a constant-current stimulator (Digitimer DS7AH, UK). For all tetanic stimulations, the stimulation intensity necessary to reach 50% of MVIC with a 0.5-s 80 Hz tetanic stimulation was used (Martin et al. 2004). Three evoked contractions of the same duration were delivered with 15 s between each contraction using the following trains: (1) 20-Hz train of 11 pulses (0.05-s interpulse interval); (2) variable-frequency train (VFT) (i.e., 2 pulses at 0.01-s plus, 10 pulses at 0.05-s interpulse interval); (3) 80-Hz train of 36 pulses (0.0125-s interpulse interval) (Trajano et al. 2013).

Knee extension force–time data were manually converted into torque-time series by multiplying force by shank length. All collected torque-time data sets were analysed using CED Spike 2 (V7.20; Cambridge Electronic Design, UK). Torque onset was manually identified and defined using the above-mentioned force-onset definition (Tillin et al. 2010) (Supplementary Fig. 2). Maximum torque in each individual 5-s trial was recorded as peak torque (PT). Peak RTD (RTD_peak_), early and late RTD (RTD_0–100_ and RTD_0–200_), and average RTD (RTD_avg_) were determined using the 1-s knee extension trials using the same formulas as for the IMTP. Once processed, the means of the 3 ‘best trials’ within each set in each testing session were used for statistical analysis. The definition of ‘best trial’ mirrored that used in the IMTP protocol.

Early EMG activity was calculated as the root-mean-square value of a 50-ms window immediately preceding torque onset (EMG_50_) (Cossich and Maffiuletti 2020) and was normalised to the *M*_max_ amplitude (EMG_50_/M) to control for potential peripheral changes (Place et al. 2007). Markers of central neuromuscular function were VA%, VL, VM, and RF and average EMG_50_/M (Maffiuletti et al. 2016), while the ratios of torques evoked by 20 Hz and 80 Hz stimulations (20:80), ratios of torques evoked by 20 Hz and variable-frequency trains (20:VFT), *Τ*_tw,p_, *t*_tw,p_ and *t*_1/2_ were used as markers of peripheral neuromuscular function (Barley et al. 2018).

### Sprint cycle protocol

Participants exercised on a Velotron cycle ergometer (Dynafit Pro Velotron; RacerMate, Seattle, USA), which was fitted with their own pedals and adjusted to their bespoke dimensions. For all participants, the ergometer was fitted with 172.5-mm Infocrank powermeter cranks (Verve Cycling, Australia) that measured left and right crank torques independently. Once-per-revolution power, cadence, and torque measurements (256-Hz analogue–digital conversion rate) were recorded via customised Infocrank data logger software (Infocrank, Australia) and stored on a mobile phone (Sony Experia Z3 Compact). The warm-up was controlled by Velotron Coaching software (RacerMate Inc., Seattle, WA, USA). During the main set, the external resistances applied to the flywheel were adjusted by manipulating the electromagnetic brake of the flywheel using the Velotron Wingate software (version 1.0; RacerMate Inc., Seattle, WA, USA). For all stationary start sprints, the crank starting position of the lead sprint leg was standardised using a wooden block at 90° (0° = Top dead centre) as this position was easiest to standardise. The lead sprint leg was selected by participants and defined as their preferred leg to start a cycling sprint.

The sprint cycle protocol commenced with participants performing a standardised 15-min warm-up. After 5-min passive rest, participants performed three 5-s sprints initiated from stationary starts against external resistances of 0.3, 0.6, 1.0 Nm kg^−1^ and two 5-s sprints initiated from rolling starts (20-s lead in) with an initial cadence of ~ 80 rpm and external resistances of 0.0 and 0.3 Nm kg^−1^. All sprints were separated by 5 min passive rest. Sprints were conducted in a randomised order in session one and standardised throughout. Vigorous verbal encouragement was provided throughout each sprint, where participants were requested to remain seated and keep their hands on the dropped portion of the handlebars.

All collected torque data were downloaded and processed using Microsoft Excel (Microsoft Corporation, USA). All RTD measurements were calculated using the average of downstrokes 2 and 3 (at a cadence of 84 ± 18 rpm) from the 1.0 Nm kg^−1^ sprint as these conditions have previously produced the greatest RTD reliability (Connolly et al. 2022). Specifically, raw torque data were manually extracted for pedal downstrokes 2 and 3 from the 1.0 Nm kg^−1^ sprint for each testing session and inserted into a custom Excel spreadsheet for calculation of RTD characteristics. Torque onset was determined visually and defined as the lowest torque value (confirmed by inspection of excel data for that pedal stroke) prior to the rapid increase above baseline to a visual peak in torque for that pedal stroke (supplementary Fig. 3). The definition for torque onset and the calculation of RTD_0–100_ and RTD_0–200_, RTD_avg,_, and RTD_peak_ mirrored those used in the knee extension test. Once individual downstrokes were processed, RTD measures were averaged for downstrokes 2 and 3.

The maximum torque produced within the subset of all downstrokes in all sprints in each session was recorded as the peak torque (*T*_peak_). The mean torque value for each pedal stroke and the cadence data were also used to develop torque–cadence (T–C) relationships. To remove non-maximal pedal strokes and construct maximal torque–cadence profiles, the following process was applied (Wackwitz et al. 2021). The data (torque, power, and cadence) for all 5-s sprints were sorted in descending order based on cadence. The torque of each effort was then compared to the prior datapoint. Given the established inverse linear association between torque and cadence (Gardner et al. 2007), if the cadence of an effort decreased without a corresponding increase in torque, the pedal stroke was deemed to be “non-maximal” and was excluded. If both the power output and cadence decreased at any point throughout a sprint, the remaining pedal strokes were excluded from the analysis as they were not considered maximal. Similar to the methods employed by Rudsits et al. (2018), the remaining data were manually filtered, so that only the highest power (and corresponding torque and cadence) per five revolution range was used to represent the relationship. Linear regression analyses were then undertaken to determine the T–C relationships (Gardner et al. 2007), with the extrapolated y-intercept of the T–C relationship being maximal torque (*T*_0_). Individual T–C relationships were modelled from 16.5 ± 2.5 (mean ± SD) data points, with *R*^2^ equal to 0.97 ± 0.02 and a standard error of the estimate of 5.20 ± 1.93 Nm.

### Statistical analysis

Processed data for each variable were averaged for the two experimental sessions before statistical analysis. Data were checked for normal distribution using a Shapiro–Wilk test. For normally distributed variables, a Pearson’s correlation (*r*) was used; however, in the event a variable was non-normally distributed, and a spearman’s rho (*ρ*) was used. The Hopkins modified Cohen’s scale was used to describe the relationships (Hopkins et al. 2009). Effects were considered as follows: < 0.1, trivial; 0.1–0.3, small (weak); 0.3–0.5, moderate; 0.5–0.7, large (strong); 0.7–0.9, very large (very strong); and > 0.9, almost perfect. All correlations were computed using Prism GraphPad (Version 9.2.0, USA). Significance was accepted as *p* ≤ 0.05.

## Results

Strong positive relationships were observed between knee extension RTD or IMTP late RFD and average EMG_50_/M (Table 2). No relationships were observed between cycling RTDs and average EMG_50_/M, or between VA% and RFD/RTD for all protocols (Table 2).

**Table 2:** Values are Pearson’s/Spearman’s correlation coefficients (*r* or *ρ*) (*p* value, 95% confidence intervals)

	KE RTD_0–100_ (Nm s^−1^)	IMTP RFD_0–100_ (N s^−1^)	Cycling RTD_0–100_ (Nm s^−1^)	KE RTD_0–200_ (Nm s^−1^)	IMTP RFD_0–200_ (N s^−1^)	Cycling RTD_0–200_ (Nm s^−1^)
VL EMG_50_/M (mV)	0.68 (< 0.01, 0.29–0.87)	0.48 (0.06, – 0.03 to 0.79)	0.51 (0.03, 0.04–0.79)	0.51 (0.03, 0.05–0.80)	0.48 (0.06, – 0.03 to 0.80)	0.50 (0.04, 0.03–0.79)
VM EMG_50_/M (mV)	0.55 (0.02, 0.09–0.81)	0.49 (0.05, – 0.02 to 0.80)	0.33 (0.18, – 0.18 to 0.70)	0.37 (0.13, – 0.13 to 0.72)	0.50 (0.05, – 0.01 to 0.80)	0.30 (0.22, – 0.21 to 0.68)
RF EMG_50_/M (mV)	0.50 (0.04, 0.02–0.79)	0.24 (0.36, – 0.30 to 0.67)	0.03 (0.91, – 0.45 to 0.50)	0.43 (0.07, – 0.06 to 0.75)	0.40 (0.13, – 0.13 to 0.75)	– 0.002 (0.99, – 0.48 to 0.48)
Average EMG_50_/M (mV)	0.65 (< 0.01, 0.25–0.86)	0.46 (0.07, – 0.06 to 0.78)	0.39 (0.10, – 0.10 to 0.74)	0.51 (0.03, 0.04 to 0.79)	0.51 (0.04, 0.00–0.80)	0.42 (0.08, – 0.08 to 0.75)
VA (%)	0.17 (0.50, – 0.32 to 0.59)	0.27 (0.31, – 0.26 to 0.68)	0.37 (0.13, – 0.12 to 0.71)	0.28 (0.26, – 0.22 to 0.66)	0.21 (0.43, – 0.32 to 0.64)	0.40 (0.10, – 0.10 to 0.73)
20:80 (Nm)	– 0.53 (0.02, – 0.80 to – 0.08)	– 0.60 (0.01, – 0.84 to – 0.14)	– 0.66 (< 0.01, – 0.86 to – 0.28)	– 0.57 (0.01, – 0.82 to – 0.13)	– 0.65 (0.01, – 0.87 to – 0.23)	– 0.59 (< 0.01, – 0.83 to – 0.16
*T*_tw,p_ (Nm)	0.35 (0.16, – 0.14 to 0.70)	0.23 (0.38, – 0.30 to 0.65)	0.70 (< 0.01, 0.35–0.88)	0.49 (0.04, 0.03–0.78)	0.34 (0.20, – 0.19 to 0.71)	0.75 (< 0.01, 0.42–0.90)
20:VFT (Nm)	0.02 (0.94, – 0.49 to 0.46)	– 0.03 (0.93, 0.53–0.49)	0.05 (0.85, – 0.51 to 0.44)	– 0.04 (0.85, – 0.51 to 0.44)	0.02 (0.95, – 0.49 to 0.52)	– 0.11 (0.67, – 0.56 to 0.39)
*t*_tw,p_ (s)	– 0.25 (0.32, – 0.65 to 0.26)	0.03 (0.92, – 0.49 to 0.53)	– 0.11 (0.66, – 0.56 to 0.39)	– 0.34 (0.16, – 0.71 to 0.16)	– 0.21 (0.44, – 0.65 to 0.33)	– 0.22 (0.38, – 0.63 to 0.29)
*t*_1/2_ (s)	– 0.16 (0.54, – 0.58 to 0.34)	– 0.20 (0.47, – 0.63 to 0.33)	– 0.54 (0.02, – 0.80 to – 0.10)	– 0.23 (0.35, 0.63–0.26)	– 0.19 (0.48, – 0.63 to 0.34)	– 0.57 (0.01, – 0.83 to – 0.13)

Associations between central and peripheral neuromuscular function measures recorded in the knee extension (KE) protocol and KE, cycling, and isometric mid-thigh pull (IMTP) rate of force/torque development from 0 to 100 ms (RFD/RTD_0–100_) and RFD/RTD from 0 to 200 ms (RFD/RTD_0–200_). Measures of central neuromuscular function include vastus lateralis, vastus medialis, and rectus femoris electromyograph amplitude to 50 ms normalised to M-wave (VL, VM, and RF EMG_50_/M), average EMG_50_/M (VL, VM, and RF EMG_50_/M/3), and voluntary activation (VA%). Measures of peripheral neuromuscular function include the ratio of torques evoked by 20-Hz and 80-Hz stimulations (20:80); ratio of torques evoked by 20 Hz and variable-frequency train (20:VFT) stimulations; peak twitch torque (*Τ*_tw,p_); time to peak twitch (*t*_tw,p_); and peak twitch half relaxation time (*t*_1/2_)

A very strong positive relationship was observed between *T*_tw,p_ and cycling RTD (Table 2). Strong negative relationships were observed between 20:80 and RFD/RTD for all protocols, and between t_1/2_ and cycling RTD (Table 2). No relationships were observed between 20:VFT or *t*_tw,p_ and RFD/RTDs for any protocol.

**Fig. 2:**
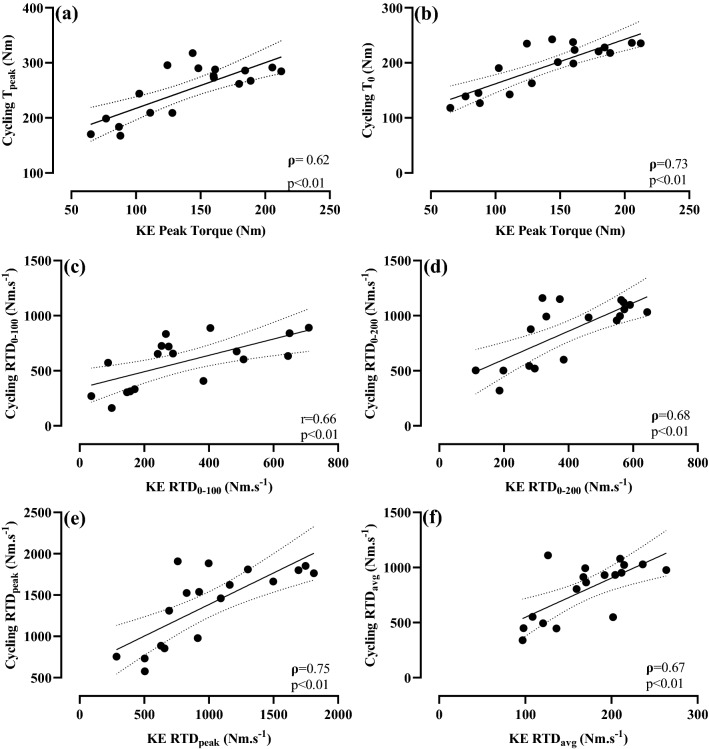
Pearson’s (*r*) or Spearman’s (*ρ*) correlation coefficients between cycling and knee extension: **a** observed cycling peak torque (*T*_peak_) and knee extension peak torque, **b** theoretical cycling peak torque (*T*_0_) and knee extension peak torque, **c** RTD from 0 to 100 ms (RTD_0–100_), **d** RTD from 0–200 ms (RTD_0–200_), **e** peak RTD (RTD_peak_), and **f** average RTD (RTD_avg_). Dotted lines: 95% confidence intervals

**Fig. 3:**
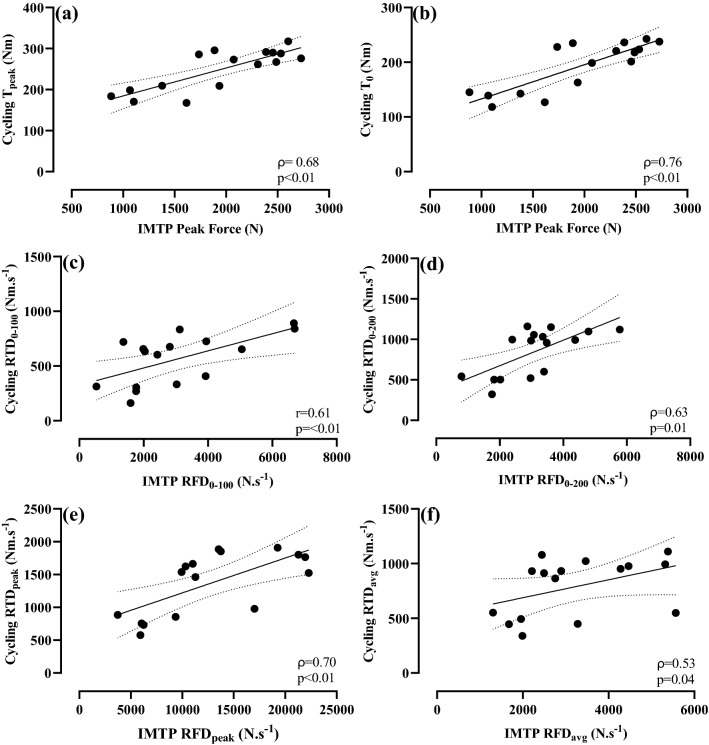
Pearson’s (*r*) or Spearman’s (*ρ*) correlation coefficients (*r*) between cycling and isometric mid-thigh pull (IMTP): **a** observed cycling peak torque (*T*_peak_) and IMTP peak force, **b** theoretical cycling peak torque (*T*_0_) and IMTP peak force, **c** RFD/RTD from 0 to 100 ms (RFD/RTD_0–100_), **d** RFD/RTD from 0 to 200 ms (RFD/RTD_0–200_), **e** peak RFD/RTD (RTD_peak_), and **f** average RFD/RTD (RFD/RTD_avg_). Dotted lines: 95% confidence intervals

Strong-to-very strong relationships were observed between knee extension, IMTP, and cycling for peak force/torque, early and late RFD/RTD, and peak RFD/RTD (Figs. 1, 2 and 3). No relationship was observed between IMTP RFD_avg_ and knee extension RTD_avg_ (Fig. 1).

## Discussion

The aims of the present study were to: (i) examine the relationships between measures of quadriceps central and peripheral neuromuscular function assessed in an isometric knee extension test and RFD/RTD in knee extension, IMTP, and sprint cycling; and (ii) investigate the relationships among RFD/RTD, and peak force/torque between knee extension, IMTP, and sprint cycling. Our key findings were: (1) while strong relationships were observed between measures of quadriceps central neuromuscular function (EMG_50_/M) and knee extension RTD and late IMTP RFD, no relationships were observed between quadriceps average EMG_50_/M and cycling RTD; (2) strong-to-very strong relationships were observed between cycling RTD and measures of quadriceps peripheral neuromuscular function (20:80, *t*_1/2_, *Τ*_tw,p_); and (3) strong-to-very-strong relationships were observed in peak force/torque, and peak, early, and late RFD/RTD across all protocols. Our findings indicate that cycling RTD may provide practitioners with an indication of a cyclist’s quadriceps *peripheral* neuromuscular function. In addition, the strong associations in peak force/torque and most RFD/RTD measures between the knee extension, IMTP, and sprint cycling indicates a level of transferability across these tasks.

In support of previous findings (Cossich and Maffiuletti 2020), we observed strong positive relationships between quadriceps early EMG activity and knee extension early RTD, with the strength of the relationship decreasing from early to late RTD (Table 2). These results taken together with those of other studies (Desmedt and Godaux 1977; Del Vecchio et al. 2019) support the notion that neural activation transmitted by motor neurons to muscles (through motor unit recruitment speed and discharge rate) may be considered as a determinant of early RTD. Of note, the correlation coefficients between quadriceps early average EMG_50_/M and early RFD/RTD decreased between the knee extension, IMTP, and sprint cycling tasks. This is not unexpected given that central and peripheral neuromuscular function was assessed within the knee extensors. However, the strong relationships observed between IMTP late RFD and average EMG_50_/M also indicate that the IMTP also has the potential to give insight into quadriceps central neuromuscular function. In contrast, RTD during cycling was not associated with central neuromuscular function (i.e., average EMG_50_/M) assessed during the knee extension. A plausible explanation for the differences in the correlation coefficients across the three tasks is due to variations in the relative contribution of the knee extensors to force/torque production between each task. This ranges from the isolation of the knee extensors for the knee extension task (Maffiuletti et al. 2016), to the multi-joint (and thus multi-muscle) isometric nature of the IMTP, to the compound movement of sprint cycling; where torque and impulse production at the crank is affected by the coordinated action of multiple lower limb muscles (Raasch et al. 1997; McDaniel et al. 2014) which activate at a different rate between pedal strokes (Rudsits et al. 2018).

In the present study, we used electrical myostimulation to obtain measurements of intrinsic muscle contractile properties without the influence of the voluntary neural drive (Andersen and Aagaard 2006). Considering the strong negative relationships observed between 20:80 Hz stimulation and RFD/RFD from knee extension, IMTP, and sprint cycling in the current study, we can speculate that RFD/RTD measurement in all protocols may provide information relating to muscular calcium concentration, sensitivity, and rate of binding to troponin (Binder-Macleod and Lee 1996; Martin et al. 2004; Binder-Macleod and Kesar 2005). Interestingly, our results showing a very strong positive relationship between *T*_tw,p_ and cycling RTD and a strong negative relationship between *t*_1/2_ and cycling RTD indicate that cycling RTD measurement may provide some insight into excitation–contraction coupling and the reuptake of calcium at the sarcoplasmic reticulum, respectively (Pasquet et al. 2003; Ørtenblad et al. 2000). Furthermore, the present results suggest that twitch parameters, such as 20:VFT and *t*_tw,p_, are not related to RFD/RTD and therefore need to be measured directly in the laboratory.

Results of the present study agree with previous research that reported strong-to-very strong relationships between knee extension and sprint cycling peak torque (Driss et al. 2002; *r* = 0.73), and between mid-thigh pull peak force and sprint cycling peak torque (Vercoe and McGuigan 2018; *r* = 0.93). These results, along with the present study’s novel findings of strong relationships in RFD/RTD_peak_, RFD/RTD_0–100_, and RFD/RTD_0–200_ between all protocols (Figs. 1, 2 and 3) indicate that a cyclist’s ability to develop force or torque rapidly (i.e., RFD/RTD) and their maximum strength expression (i.e., peak force/torque) was transferable when measured during knee extension, IMTP, or sprint cycling. Specifically, cyclists who had a faster RTD or greater peak torque in knee extension also produced superior RFD/RTD and peak force/torque in both the IMTP and sprint cycling protocols. One notable exception to this was RFD/RTD_avg_ where no relationship was observed between knee extension and IMTP (Fig. 1), and thus, caution should be applied if using this measure to determine relationships between these tasks. Of note, cycling *T*_peak_ and *T*_0_ displayed similar relationships with knee extension peak torque or IMTP peak force (Figs. 2, 3), indicating that the measurement of knee extension peak torque and IMTP peak force provides a similar indication into sprint cycling peak torque generation expressed as either of these parameters.

It is important to note that this study was conducted when participants were in a rested state and thus, caution should be taken when applying the relationships herein for longitudinal training monitoring. Since relationships do not necessarily imply “cause and effect”, future training and interventional studies should be conducted to determine relationships between the training-induced changes in peak force/torque and RFD/RTD. To determine the potential practical utility to practitioners, consideration should also be directed at determining the sensitivity of each variable to detect training-induced changes.

As with any investigation, there are limitations to our work that should be considered. First, we acknowledge that the sampling rate used to measure the RTD is lower than the ≥ 1000 Hz proposed by Thompson (2019) to accurately assess RTD of ≤ 50 ms or RTD_peak_. While we used a lower sampling rate of 256 Hz for the cycling protocol, Thompson (2019) was assessing an isometric knee extension task in which faster RFD is expected given the significant 'impact' that occurs between the shin and load cell at commencement of the contraction. Conversely, the cycling protocol involved a multi-joint, dynamic cycling task where lower impact and much slower RTD is expected. There are multiple potential reasons for a slower RTD in cycling, including a greater movement complexity that requires significant between-segment muscle activation coordination, increased total compliance resulting from a greater total muscle and joint tissue volume, and the variability introduced by the muscles being activated prior to the rapid, propulsive downstroke that may introduce force application variability (Van Cutsem and Duchateau 2005). For these reasons, a lower sampling rate may be sufficient for accurate RTD measurement in cycling. Of note, the Infocrank powermeter used in the present study has one of the highest torque sampling rates available commercially. Therefore, the findings herein are applicable practically in the field. Further studies may more specifically examine the effect of sampling rate on RTD magnitude and reliability in sprint cycling, which would help with future decision-making around the optimum methodology to use when assessing RTD in sprint cycling. Next, we acknowledge that EMG was only assessed in the knee extension protocol and not in the IMTP or cycling tests, which may have strengthened the conclusions that could be drawn from our data. Accordingly, future studies could measure EMG in each protocol to determine the associations between RFD/RTD and EMG in the same protocol. Finally, the present study focussed on analysing cycling RTD for downstrokes two and three of a sprint (at a cadence of 84 ± 18 rpm). It should be acknowledged that peak power commonly occurs at 120–130 rpm (Martin et al. 2007), and therefore, the cadences in which we measured RTD are not the cadences at which this variable has the greatest potential impact on power production during sprint cycling. To this end, future studies should focus on examining cycling RTD at higher cadences (> 120 rpm).

## Conclusions

To the authors’ knowledge, this is the first study to examine the relationship between measures of quadriceps central and peripheral neuromuscular function obtained during an isometric knee extension task and early and late RFD/RTD measured in the IMTP and sprint cycling. Another novel aspect of this study was the investigation of the relationship between RFDs obtained from single-joint and whole-body isometric tests and RTD produced during sprint cycling using well-trained cyclists. Results of the present study provide information to assist decision-making around protocol selection for practitioners and researchers interested in getting an indication of a cyclist’s quadriceps central or peripheral neuromuscular function through the measurement of RFD or RTD. In addition, our findings of the relationships in peak force/torque, and peak, early, and late RFD/RTD in knee extension, IMTP, and sprint cycling provide practitioners and researchers with information about the transferability across measurements within these tasks.

## Supplementary Information

Below is the link to the electronic supplementary material.

Supplementary file1 (PDF 199 KB)

## Data Availability

All data generated or analysed during this study are included in this published article (and its supplementary information files).

## Abbreviations

EMG: Electromyogram
IMTP: Isometric mid-thigh pull
$M_{\max }$: Maximum muscle compound action potential
MVIC: Maximal voluntary isometric contraction
NMF: Neuromuscular function
PF: Peak force
RFD: Rate of force development
RF: Rectus femoris
RTD: Rate of torque development
${\text{RTD}}_{{{\text{peak}}}}$: Peak rate of torque development
${\text{RTD}}_{{{\text{avg}}}}$: Average rate of torque development
$T_{{{\text{peak}}}}$: Observed peak torque
$T_{0}$: Theoretical peak torque
$t_{{\text{tw,p}}}$: Time to peak twitch
$T_{{\text{tw,p}}}$: Peak twitch torque
$t_{1/2}$: Peak twitch half relaxation time
VA: Voluntary activation
VL: Vastus lateralis
VM: Vastus medialis
