# Supplementary material for: Sprint cycling rate of torque development associates with strength measurement in trained cyclists
Source: Eur J Appl Physiol. 2023 Feb 10;123(6):1215–27. doi: 10.1007/s00421-023-05143-1 (PMC10191994; doi:10.1007/s00421-023-05143-1)
Supplement: Supplementary file 1 — Supplementary file1 (PDF 199 KB) [file 421_2023_5143_MOESM1_ESM.pdf]

Supplementary figures:

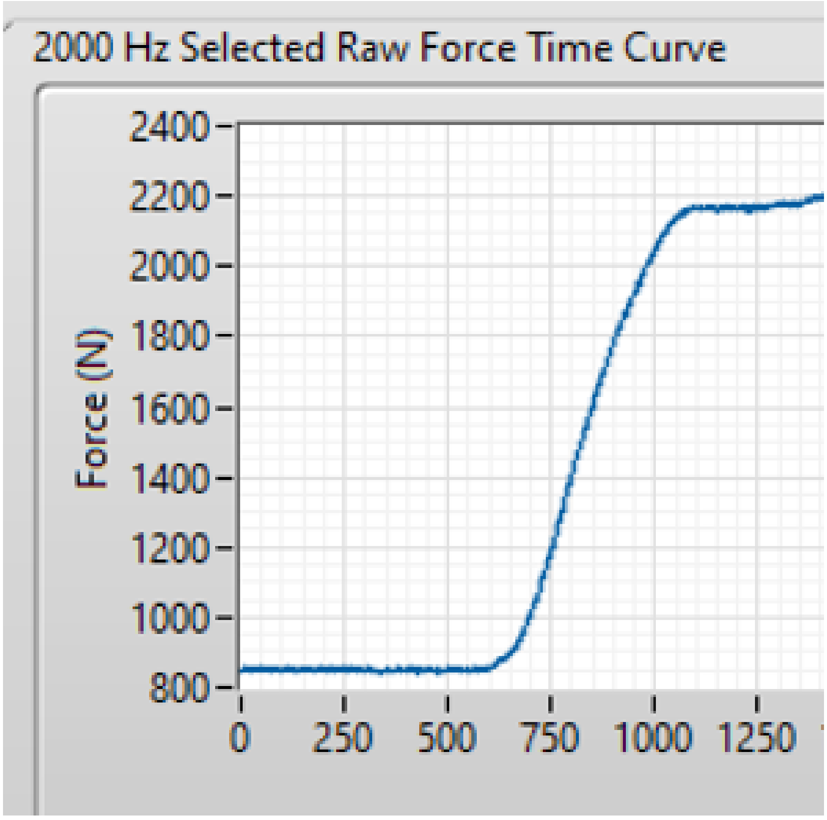

Supplementary figure 1: An example of the force-onset and rise in force during a 1-s isometric mid-thigh pull trial.

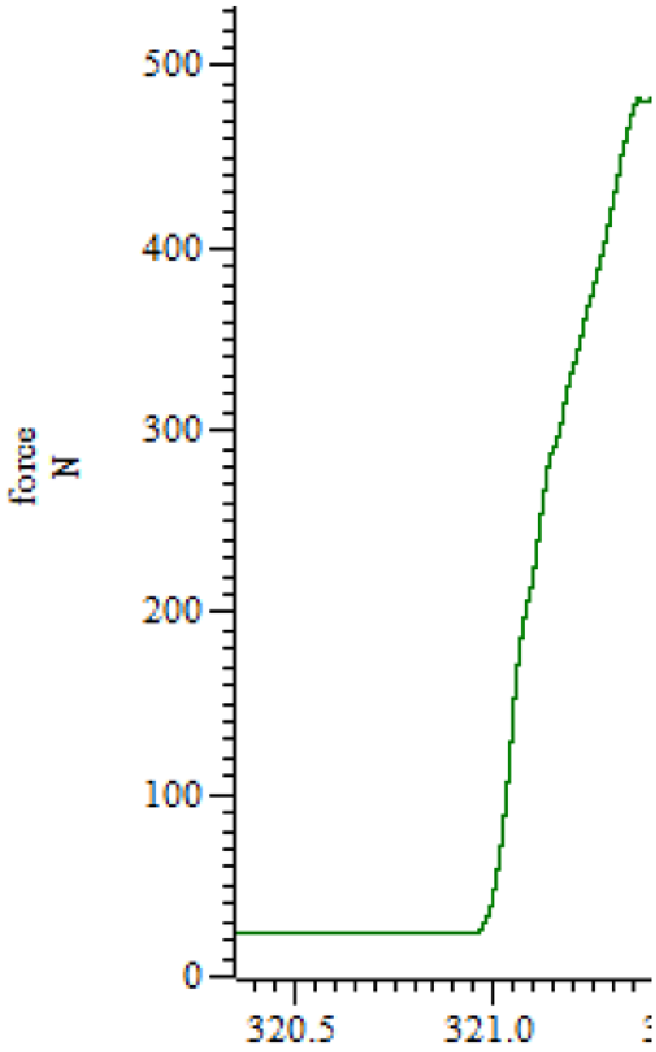

Supplementary figure 2: An example of the force-onset and rise in force during a 1-s knee extension trial.

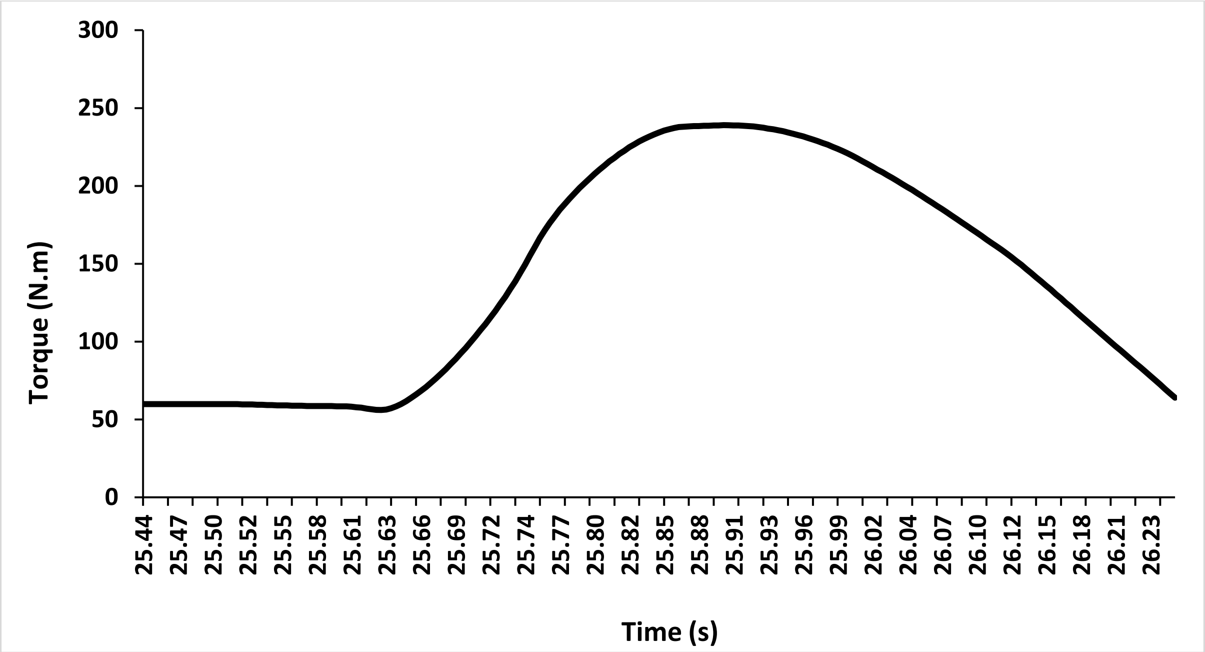

Supplementary figure 3: An example of the sprint cycling torque-time data from 1 pedal stroke.
